# Impact of Dietary Fiber on Inflammation in Humans

**DOI:** 10.3390/ijms26052000

**Published:** 2025-02-25

**Authors:** Stefan Kabisch, Jasmin Hajir, Varvara Sukhobaevskaia, Martin O. Weickert, Andreas F. H. Pfeiffer

**Affiliations:** 1 Department of Endocrinology and Metabolism, Campus Benjamin Franklin, Charité University Medicine, Hindenburgdamm 30, 12203 Berlin, Germany; 2 Deutsches Zentrum für Diabetesforschung e.V., Geschäftsstelle am Helmholtz-Zentrum München, Ingolstädter Landstraße 1, 85764 Neuherberg, Germany; 3 Warwickshire Institute for the Study of Diabetes, Endocrinology and Metabolism; The ARDEN NET Centre, ENETS CoE; University Hospitals Coventry and Warwickshire NHS Trust, Coventry CV2 2DX, UK; 4 Centre of Applied Biological & Exercise Sciences (ABES), Faculty of Health & Life Sciences, Coventry University, Coventry CV1 5FB, UK; 5 Translational & Experimental Medicine, Division of Biomedical Sciences, Warwick Medical School, University of Warwick, Coventry CV4 7AL, UK

**Keywords:** dietary fiber, cellulose, inulin, resistant starch, ß-glucans, pectin, inflammation, CRP, IL-6, IL-1, TNF-alpha

## Abstract

Cohort studies consistently show that a high intake of cereal fiber and whole-grain products is associated with a decreased risk of type 2 diabetes (T2DM), cancer, and cardiovascular diseases. Similar findings are also reported for infectious and chronic inflammatory disorders. All these disorders are at least partially caused by inflammaging, a chronic state of inflammation associated with aging and Metabolic Syndrome. Surprisingly, insoluble (cereal) fiber intake consistently shows stronger protective associations with most long-term health outcomes than soluble fiber. Most humans consume soluble fiber mainly from sweet fruits, which usually come with high levels of sugar, counteracting the potentially beneficial effects of fiber. In both observational and interventional studies, high-fiber diets show a beneficial impact on inflammation, which can be attributed to a variety of nutrients apart from dietary fiber. These confounders need to be considered when evaluating the effects of fiber as part of complex dietary patterns. When assessing specific types of fiber, inulin and resistant starch clearly elicit anti-inflammatory short-term effects, while results for pectins, beta-glucans, or psyllium turn out to be less convincing. For insoluble fiber, promising but sparse data have been published so far. Hypotheses on putative mechanisms of anti-inflammatory fiber effects include a direct impact on immune cells (e.g., for pectin), fermentation to pleiotropic short-chain fatty acids (for fermentable fiber only), modulation of the gut microbiome towards higher levels of diversity, changes in bile acid metabolism, a differential release of gut hormones (such as the glucose-dependent insulinotropic peptide (GIP)), and an improvement of insulin resistance via the mTOR/S6K1 signaling cascade. Moreover, the contribution of phytate-mediated antioxidative and immune-modulatory means of action needs to be considered. In this review, we summarize the present knowledge on the impact of fiber-rich diets and dietary fiber on the human inflammatory system. However, given the huge heterogeneity of study designs, cohorts, interventions, and outcomes, definite conclusions on which fiber to recommend to whom cannot yet be drawn.

## 1. The Long-Term Benefits of Dietary Fiber in Past and Present

Modern man has managed to evolve in parallel to his diet within the course of a few thousand years. Humans have always consumed smaller or larger portions of meat, even though in most regions of the world nowadays venison, bugs, larvae, and worms have been completely replaced by pork, poultry, beef, and mutton with a more striking amount of consumed meat. Dairy has only been introduced in the last few millennia, again with a remarkable increase in world-wide demand. Plant-based foods have largely changed in their individual variety and composition. The Paleolithic diet, characterized by gathered grains, seeds, fruits, berries, roots, leafy plants, and nuts, has turned into an often highly processed collection of vaguely plant-based products, where fruits and most importantly cereals have gained impact. Nuts and berries have become a modern-day luxury product, for several reasons. Also, food plants themselves have changed. From the beginning of agriculture, crop selection has favored sorts and variations, which provide a higher yield and/or sweeter taste. The biological consequences for cultivated plants are a reduction in genetic diversity and—concomitantly—a continuous decrease in nutritional quality, before and after food processing. Contemporary breeds and hybrids are optimized for size, color, and sweet taste while lacking minerals, vitamins, polyphenols, and fiber [1,2,3]. Modern techniques of food processing—used all over the world—cause an additional deficit in all these essential micronutrients. In the case of cereal products, it leads to a loss of 60–75% of cereal fiber when turning whole grain into white flour [4]. All unhealthy diets share a common feature that is distinctive in comparison to all healthy diets: low intake of dietary fiber. The average Stone Age diet contained lots of fiber; estimates range from at least 50 to 100 g per day [5]. Daily fiber intake has dropped to 20 g or even below that in the mid-20th century and has slowly increased by very few grams in all social strata for the past decades [6,7,8].

Epidemiological evidence shows a strong impact of dietary fiber on human health. Low intake has been identified as a major risk factor for a series of late-onset chronic disorders, often beginning with obesity and leading to type 2 diabetes mellitus (T2DM) [9,10,11,12], hypertension, dyslipidemia, non-alcoholic fatty liver disease (NAFLD) [13,14], and, finally, to cardiovascular disease (CVD) and premature (often CVD-related) death [15,16,17,18,19]. Reduced fiber intake is also connected to certain types of cancer of the gastrointestinal tract [20,21]. All these protective associations are predominantly shown for whole grain and insoluble fiber [21,22,23].

Furthermore, fiber intake has been shown to reduce the risk of mortality due to inflammatory and infectious diseases in general [24], but also to specific disorders of this group: protective association is described for chronic obstructive pulmonary disease [25,26] and asthma [27]. An association between fiber intake and various chronic autoimmune inflammatory disorders remained insignificant in a Danish cohort study, which found the trendwise linkage to be eliminated after adjustment for confounders [28]. However, for other diseases with primarily inflammatory courses of action, dietary fiber shows beneficial associations: Crohn’s disease (but not ulcerative colitis), which is less common among persons with a high intake of fruits and vegetables [29]; and rheumatoid arthritis, which is improved by the fiber-rich traditional Mediterranean diet [30,31].

**Figure 1:**
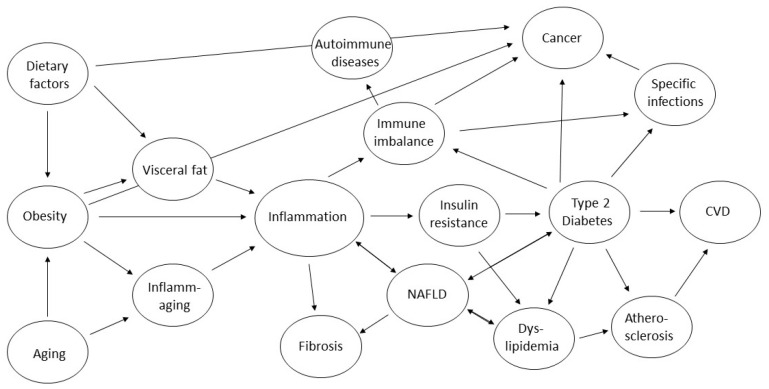
Involvement of inflammation in diet, obesity, aging, and long-term outcomes.

Almost all very common life-threatening long-term (co-)morbidities—ranging from metabolic and cardiovascular disorders to cancer and degenerative disorders—share chronic inflammation as a pathophysiological component (Figure 1). In the case of NAFLD, cancer, or some infectious diseases, reasons for the development of a sustained inflammatory reaction are quite specific and predominantly develop in a certain organ. NAFLD is promoted by an imbalance of lipid storage, fat oxidation, and de novo lipogenesis in the liver. Cancer is partially facilitated by toxins with local activity and agents, which increase the production of reactive oxygen species (ROS). Degenerative disorders gain activity by regional metabolic dysfunction and mechanic destruction. Infectious diseases require a certain germ of any kind but may proliferate when the immune system is unbalanced. However, all these examples of rather localized inflammation are often also associated with systemic inflammation. The typical common precursor of NAFLD, CVD, and cancer—Metabolic Syndrome—facilitates the bidirectional development of a continuously elevated alarm situation of several inflammatory pathways, which is further supported by aging (inflammaging) [32]. The term “inflammaging” amalgamates the decline in adaptive immune response and a compensatory or reactive increase in continuous activity of the unspecific innate immune system during aging. This state leads to a higher susceptibility to certain infections due to lower specific protection, but also to inflammation-driven aging itself, as the flourishing subclinical inflammation contributes to vascular damage, insulin resistance, and fibrosis, i.e., inflammaging is both inflammation by aging and aging by inflammation, both of which can be targeted by a healthy diet [33,34]. A main source of the pro-inflammatory mediators is the visceral adipose tissue (VAT), which shows increased volume and activity in the course of obesity and T2DM; a main target of this inflammation, progressing with age, is blood vessels.

In clinical studies, measures of inflammation include the amount of visceral fat, but also a variety of circulating blood parameters. Besides the leukocyte count, several classes of measurable peptides describe the inflammatory state. C-reactive protein (CRP) is the most common acute-phase protein involved in opsonizing potential immune cell targets. A whole group of proteins, less related to actual inflammatory action, respond in parallel (positively) or inversely (negatively) to inflammation processes. Among these, fibrinogen and ferritin are sometimes evaluated as additional measures of inflammation. Similar to CRP, their hepatic production is strongly influenced by broadly acting immune mediators such as interleukins-1, -6, and -8 (IL-1, IL-6, IL-8); monocyte-derived chemoattractive protein (MCP), tumor necrosis factor alpha (TNF-alpha); and cell-adhesion molecules (CAM), which include selectins, cadherins, and integrins. There are also specific anti-inflammatory players such as the interleukins 1 and 10 (IL-1-beta, IL-10) [35]. Learning from this first overview, heterogeneity of potential outcomes already predicts a certain heterogeneity in study results, limiting the generalizability of clinical recommendations.

## 2. Effects of Certain Fiber-Rich Diets on Inflammatory Outcomes in Cohort Studies and RCTs

Fiber-rich diets are often characterized by a higher load of total carbohydrates but a lower glycemic index (GI) and glycemic load (GL). This is true for healthy low-fat diets in general, but also for the more strictly defined vegetarian and vegan diets. All of those diets provide more than 50 kcal% by carbohydrates, which needs to be considered when assessing long-term outcomes. Observational studies show an increased mortality associated with both low-carbohydrate and carbohydrate-rich diets with a variable optimum for carbohydrate proportion [36,37,38]. On the other hand, as a result of the first large observational studies describing an association between fat intake and CVD risk, the high-carb low-fat diet has been recommended for decades [39,40]. Its benefits have been attributed to low-fat content rather than high content of fiber, leading to a shift in world-wide dietary recommendations that focused on fat, but neglected the role of sugars, fiber, and glycemic impact [41,42]. Low-fat diets, especially when limiting the intake of saturated fat, have been consistently shown to lower levels of CRP as the most commonly measured inflammatory marker [43].

Low-carb and ketogenic diets are generally considered beneficial for weight loss and metabolically associated inflammation [44,45]. However, a trend towards less favorable or even detrimental development of inflammation was reported in several, but not the majority of, RCTs. Such lack of benefit is seen despite equal-to-superior weight loss [46,47,48] or in the absence of weight loss [49]; furthermore, patients with low baseline levels seem to be particularly susceptible to increasing inflammation under low-carb conditions [46,50]. However, some possibly confounding food products and components need to be considered as pivotal players (see next chapter).

While low-fat diets *can* show a low GI or low GL, actual total carbohydrate restriction reduces the total intake of all saccharides. Unfortunately, the GI concept only addresses glucose and its digestible polymers, but not fructose or galactose, which also contribute to the metabolic damage of unhealthy diets. Low GI may result from high amounts of fiber, irrespective of fructose content. Therefore, “low GI” or “low GL” diets do not globally improve inflammation. A meta-analysis on 28 RCTs (2961 patients) did not observe a significant benefit on CRP, TNF-alpha, or IL-6 [51], while another systematic review with meta-analysis (SRMA) pooling 1617 diabetes patients from 28 RCTs reported lower CRP levels under a low GI regime [52]. A different SRMA on RCTs with T2DM or GDM patients did not replicate the effect on CRP but found a significant reduction in IL-6 [53]. Replacing different types of sugar for another does not seem to affect CRP [54,55].

In cohort studies, vegan and vegetarian subjects are predominantly female and younger. They are also characterized by a higher socioeconomic status, lower rates of smoking, lower alcohol intake, and higher levels of physical activity [56,57,58]. Therefore, cross-sectional or non-randomized prospective data on the vegetarian/vegan diet, showing lower levels of CRP, fibrinogen, and leukocyte count [59], cannot be interpreted without considering these relevant confounders. While plant-based diets appear to be superior with respect to CVD and cancer risk in epidemiological studies [56,57], intervention studies rate them as moderately effective for glucose control in T2DM [60] but poorly effective for antihypertensive treatment or normalization of lipid levels [61,62] despite supporting weight loss [63]. Still, they seem to improve inflammation parameters more strongly than control diets, which could be attributed to their consistently exceptionally high content of vitamins and fiber and their low amount of iron [64,65]. As for vitamins and fiber, an ideal omega-6/omega-3 ratio with low contents of sugar and purines is possible, but not mandatory for vegetarian diets in common practice; ergo, these factors may explain the anti-inflammatory properties of plant-based diets if present, but achieving those goals depends on health self-consciousness and may still be accommodated by nutritional deficits [66,67].

The traditional Mediterranean diet resembles a slightly carbohydrate-reduced dietary pattern, providing roughly 35–45% of its energy by mono-, oligo-, and polysaccharides [68]. By tradition and medical recommendation, high-glycemic carbohydrate sources such as sugary beverages, cereals, and pastries are discouraged. Typical carbohydrate sources are low-to-moderate glycemic grain products [69,70], legumes, nuts, fruits, and vegetables; ergo, a concomitantly high load of fiber. Further emphasized elements are extra-virgin olive oil, fatty fish, preferred intake of white meat rather than red meat, and the consumption of red wine. These components automatically reduce carbohydrate intake without the need to focus on that aim. The traditional Mediterranean diet receives praise from observational studies for its association with lower risks for CVD, cancer, and T2DM, all of which are confirmed by a set of partially long-term RCTs, such as the PrediMed study [71]. In interventional settings, the traditional Mediterranean diet reduces CVD risk and ranks among the best-studied diets to improve all aspects of the Metabolic Syndrome [71,72,73]. It is also highly effective in reducing inflammation [74,75], more so than the low-fat diet [76]. There is also evidence for reduced risk of breast and several kinds of gastrointestinal cancer [77,78,79]. Clinical evidence for benefits is mostly seen in studies conducted in Mediterranean countries [73], possibly showing a limited acceptance in other regions (such as Germany, Australia, or the UK) [80,81,82]. Attributing the metabolic benefits of the traditional Mediterranean diet to a certain food component is difficult. High amounts of vitamins, minerals, essential and non-essential unsaturated oils, and fiber are paralleled by low intake of sugar, iron, and purines. Every component contributes to the overall effect. Thus, we need to investigate the impact of certain food groups in order to elucidate their individual effect on metabolism and—specifically—systemic inflammation.

The DASH diet is specifically composed to reduce hypertension [83,84] but also addresses insulin resistance, dyslipoproteinemia, and inflammation. In cohort studies, adherence to the DASH diet is associated with reduced levels of CRP [85,86,87,88]. Several RCTs have confirmed this effect in interventions [89,90,91], while—for reasons unknown—others failed to do so [92,93].

In conclusion of this section, high-fiber diets are certainly healthy—irrespective of macronutrient composition. But the question of to what extent specific high-fiber foods are pivotal or neutral in effect and whether other dietary components are the actual gamechangers to tackle chronic inflammation can only be answered by more specific studies on selected high- and low-fiber foods investigated in isolated interventions.

## 3. Impact of Specific High- and Low-Fiber Foods on Inflammatory Outcomes

High-fiber diets are characterized by an abundance of whole grains, nuts, seeds and legumes, fruits, and/or vegetables. Low-fiber diets are often rich in (red processed) meat, dairy, and processed sources of carbohydrates.

### 3.1. Whole Grain

In particular, whole-grain products are widely investigated for their widespread benefit on long-term risks such as CVD, T2DM, cancer, and infectious diseases [94]. A recent meta-analysis of RCTs (9 trials, *n* = 838) has demonstrated a consistent anti-inflammatory effect of dietary interventions with whole grains. CRP was particularly reduced in studies with overweight or obese adults and when using dosages of above 100 g per day. IL-6 decreased significantly without subgroup specificity. TNF-alpha and IL-1β did not differ between the treatments [95]. The borderline benefit for CRP and IL-6 was confirmed by later replication SRMAs [96,97,98], but the effects seemed to be driven by two single RCTs [99,100]. Short-term interventions appear to be less efficient in reducing inflammatory parameters [101].

### 3.2. Nuts, Seeds, and Legumes

The PrediMed study showed a benefit on CVD from traditional Mediterranean diets with olive oil or walnuts. Overall mortality was only reduced by olive oil and not nuts. This is paralleled by findings on CRP, which decreased in the EVOO group (extra-virgin olive oil), but not the nuts group. On the other hand, IL-6, VCAM, and ICAM decreased in both Mediterranean groups but increased in the low-fat control group [102]. In a first meta-analysis, the impact of tree nuts on CRP, TNF-alpha, IL-6, IL-10, E-selectin, VCAM, and ICAM, pooled and partially separated for pistachios and almonds, was found to be entirely insignificant [103]. A later SRMA came to the same conclusion when investigating these outcomes [104].

Flaxseed interventions seem to beneficially affect the inflammatory state. HsCRP and TNF-alpha, maybe also IL-6, were found to decrease more strongly in groups of people receiving treatment with flaxseed or flaxseed derivatives. However, the SRMAs on 32 trials reported strong heterogeneity of the results, attributable to the type of study, type of intervention, overall study quality, patients’ age, and BMI [105]. Effects on CRP, IL-6, and VCAM, but not TNF-alpha, ICAM-1, or selectin were reported in another SRMA on flaxseed supplementation [106]. Other SRMAs on particular cohorts specifically reported significant results on CRP in obese subjects or patients with coronary artery disease [107,108]. One has to consider that flaxseed contains healthy oil, which itself could decrease inflammatory activity. A recent SRMA describes limited effects on IL-6, but no other major parameter of inflammation [109]. Therefore, pinpointing the effects of flaxseed on its fiber content rather than any other flaxseed component is not entirely possible. Consistent anti-inflammatory effects on CRP levels are described for natural soy products [110] and legumes [111]. In contrast, isolated soy isoflavones do not improve the inflammatory status [110,112], highlighting legume protein or fiber as the putative main components counteracting inflammation.

### 3.3. Fruits and Vegetables

Surprisingly, in contrast to whole grain or insoluble fiber, soluble fiber (from fruit and vegetables) shows weaker or insignificant associations with the risk for T2DM and infectious or inflammatory disorders [10,24,25,26,27,113]. Within whole grain, discrimination of insoluble and soluble fiber has not been investigated in cohort studies due to methodological difficulties. In studies comparing interventions with whole grain vs. fruits and vegetables, providing the same total amount of fiber, study groups receiving fruits and vegetables had a smaller benefit with respect to their inflammatory status [114]. For specific vegetables, minor effects on CRP or IL-6 were reported in SRMAs [115]. As their comparison is based on complete food, but not fiber, the difference might be attributable to sugar load as well.

### 3.4. Other Relevant Foods Within Fiber-Rich Diets

Observational studies have linked several other foods or food components with T2DM, CVD, and cancer risk, which may indirectly reflect fiber intake. This covers sugar, red (processed) meat, and coffee. High intake of sugar-sweetened beverages is associated with a pro-inflammatory proteome signature [116]. However, no single type of sugar seems to be exceptionally detrimental [54,55]. Cohort studies are linking the intake of high amounts of meat, in particular red and processed meat, with inflammatory and cardiometabolic long-term outcomes [117,118], but none of the few well-controlled RCTs comparing red and white meat in an isocaloric fashion evaluated inflammation [119,120,121,122,123,124]. Other RCTs on red meat vs. non-meat high-protein control (dairy, legumes) are inconclusive with respect to CRP, TNF-alpha, and leukocyte count [125,126,127,128,129], and whey as a control protein seems to improve CRP levels [130]. Coffee consumption is considered as beneficial from the point of view of observational studies, while RCTs did not assess inflammation parameters [131,132,133].

Similarly to the limitation of studies on complex high-fiber diets (Section 2), even research on specific high-fiber foods does not provide the final clarification about the impact of certain types of fiber on metabolic and related inflammatory outcomes. The observed beneficial or detrimental effects could also be explained by other components of high-fiber or low-fiber foods. These confounders are discussed in the following section.

## 4. Which Confounding Nutrients of Fiber-Rich Diets Trigger or Antagonize Inflammation?

Even though specific fiber-rich foods seem to reduce inflammation, some effects may be attributed to other nutrients such as healthy oils, vitamins, and minerals. Products with a high glycemic index are low in all these components but high in energy-dense starch and saccharides, which promote visceral obesity, insulin resistance, and inflammation in mechanistic trials [134,135,136], confirmed by meta-analyses comparing sugars with complex carbohydrates [137].

Low-carb diets, which avoid both digestible carbohydrates and fiber, are typically rich in protein, fat, and iron. For high-protein diets, clinical evidence indicates an anti-inflammatory effect under hypo- and isocaloric conditions [138,139,140,141,142] but possibly increased inflammation in hypercaloric situations. Saturated fat is labeled as a pro-inflammatory nutrient. Early epidemiological studies have linked excess intake of saturated fat with CVD and premature death [39,40]. Today’s SRMAs of cohort studies and RCTs report a small-to-absent impact of saturated fat on CVD and T2DM risk [143,144], as well as on inflammation [145].

Short-chain fatty acids (SCFAs) are both an original part of our diet and a secondary product due to GI fermentation of fiber, polyols, and excess digestible carbohydrates. SCFAs—also a part of fermented foods—may reduce insulin resistance, but also inflammation [146,147].

Mono-unsaturated fatty acids, as predominantly found in olive oil, were not found to decrease CRP in several studies [148,149,150,151], while showing a benefit in others [152,153]. A recent SRMA reports a relevant impact of olive oil on IL-6 and, to a smaller extent, on TNF-alpha and CRP [154].

Polyunsaturated fatty acids (PUFAs) are classified as omega-6 and omega-3 PUFAs. Omega-6 PUFAs are considered precursors of arachidonic acid, leukotrienes, and pro-inflammatory prostaglandins. Omega-3 PUFAs, the natural antagonists of omega-6 PUFAs, are metabolized to anti-inflammatory class-three prostaglandins. Therefore, a low omega-6/omega-3 ratio—as found in most high-fiber diets—has been considered optimal for metabolic health in the past decades. As omega-6 PUFA-derived lipoxins and omega-6 PUFA linoleic acid are more and more seen as rather anti-inflammatory mediators, the omega-6/omega-3 ratio as an indicator of inflammatory balance becomes less valid [155,156]. Achieving a low ratio in RCTs was found to possibly reduce IL-6 and TNF-alpha in some groups of patients, while CRP was not affected [157]. Supplementing conjugated linoleic acid (omega-6) consistently increased CRP and TNF-alpha [158,159], but not IL-6 [160].

In contrast to conventional omega-3 PUFAs (such as alpha-linolenic acid (ALA)), which are found in all plants, long-chain omega-3 PUFAs such as eicosapentaenoic acid (EPA) and docosahexaenoic acid (DHA) are solely found in marine products such as fatty fish and algae. In a 2018 SRMA, ALA was found not to decrease TNF-alpha, IL-6, VCAM, and ICAM in the overall selection of 25 supplement studies but possibly even to increase CRP levels, specifically conducted in healthy subjects [161]. This beneficial effect of omega-3 PUFAs on CRP is also seen in patients undergoing hemodialysis [162]. For marine n3-PUFAs, a meta-analysis of 68 RCTs reports a clear reduction in CRP, TNF-alpha, and IL-6, similarly in healthy persons and patients with chronic diseases of various origins [163]. EPA and DHA also consistently reduced CRP in eight studies with T2DM patients [164]. In a single study in T2DM patients, IL-2 was reduced, while IL-6 remained unchanged [165]. However, recommendations for fish oil supplementation are still cautious, as there is no consistent evidence for CVD prevention, but a potentially increased risk for atrial fibrillation [166].

Antioxidative vitamins (C, E), a series of polyphenols, and other complex plant substances from high-fiber diets are linked to ROS cleavage and, therefore, limited inflammation [167]. As for most of the non-vitamin compounds, bioavailability is low or unknown, and the clinical relevance of these substances for any cell beyond the gut layer is often disputed [168,169].

Fiber-rich diets are characterized by higher loads of zinc, magnesium, and selenium, all of which are described as potentially anti-inflammatory from RCT SRMAs [170,171,172].

Dietary iron, especially in the form of haeme iron and animal ferritin, is debated as an inducer of ROS and inflammation [118,173]. Purines and uric acid, mostly found in meat, alcoholic beverages, legumes, and some leafy vegetables, are both potential mirrors and stimuli of systemic inflammation, and induce the localized immune reaction in the case of gout [174,175]. There are no intervention studies testing the inflammatory impact of iron or purines.

The beneficial effect of fiber might also be attributed to a typical by-product of plant-based indigestible carbohydrate phytate. This compound is a common partner of fiber in cereals, seeds, and legumes. Phytic acid slows down starch digestion by a considerable magnitude in a variety of foods [176,177,178]. It is also able to chelate certain types of metal ions, namely iron, calcium, magnesium, and zinc [179]. By this, it contributes to malnutrition with necessary minerals and protects the body from an overload of potential ROS stimuli, in particular iron [180]. Excess levels of iron are linked to type 2 diabetes and colon cancer, potentially driven by local and/or systematic ROS-driven inflammation [181,182]. This protective effect might also extend to other toxic substances that are contained in the gut lumen and excreted by feces. Independently of iron chelation, phytic acid appears to reduce oxidative stress in acute in vitro stimulation experiments [183]. In addition, phytate seems to have an intrinsic effect on the gut cell cycle, triggering apoptosis in cancer cells when administered in combination with butyrate [184] and reducing cancer incidence in rats after chemical tumor induction [185]. Feeding phytate to rats also improved gut microbiome diversity and production of SCFAs, leading to reduced levels of pro-inflammatory cytokines [186]. This anti-inflammatory effect is at least partially located in the gut cells themselves, mediated via the nuclear factor kappa B (NFκB) and/or the mitogen-activated protein kinase B (MAPK-B) [187,188,189]. Epidemiologically, high intake of phytate is associated with lower levels of CRP [190]. A 6-week intake of phytate in postmenopausal women led to a reduction in iron, ferritin (an acute-phase protein), and transferrin saturation, but did not alter CRP levels [191].

The reviewed publications in this chapter are often supplementation studies. This selection follows the intention to pinpoint specific compounds rather than complex foods to potential effects on inflammatory regulation. It is by no means a recommendation to actually broadly consume these compounds as supplements, in particular as some are advertised as beneficial, even though high-quality research tells otherwise. Any compound that is evidently healthy should be preferably consumed as part of an overall healthy diet.

## 5. Interventional Evidence for Anti-Inflammatory Properties of Specific Types of Fiber

Globally, higher intake of fiber leads to a significantly stronger reduction in CRP levels as shown by recent large RCT meta-analyses on the treatment of patients with diabetes or critical illness [192,193]. However, the heterogeneity of cohorts, treatments, intervention durations, and, therefore, results demands a stratification. Anti-inflammatory effects seem to require a certain state of metabolic impairment, as healthy cohorts may show a bottom effect, leaving studies in normal-weight and/or normoglycemic persons apparently unsuitable to demonstrate an impact on inflammation [194]. In patients with defined inflammatory disorders—such as rheumatoid arthritis—unspecific uncontrolled fiber treatments improved cytokine profiles, markers of bone erosion, and symptom load [195,196]. In COPD patients, sugarcane fiber improved their quality of life despite unaltered symptoms [197]. When talking about fiber, its chemical diversity needs to be considered, as it largely determines the specific health impact on humans [198,199]. Table 1 provides an overview of all common fiber types according to their chemicophysical properties.

### 5.1. Insoluble Fiber

Apart from studies on whole grain, RCTs investigating specific types of insoluble fiber (cellulose, hemicellulose, certain arabinoxylans, lignin) are sparse. The “Protein and Fiber in Metabolic Syndrome” study primarily investigated the effects of insoluble cereal fiber on insulin sensitivity over 18 weeks. Besides their findings on glucose metabolism, there were no significant differences in VAT, CRP, or PAI-1 [200]. The two-year Optimal Fibre Trial on Diabetes Prevention used the same supplement. In their secondary results, leukocyte count, but not VAT and CRP, were significantly stronger in the fiber group, especially in obese patients and those with combined glucose impairment (vs. isolated glucose intolerance) [201,202,203,204].

### 5.2. Prebiotic (Fermentable) Fiber and Synbiotics

Prebiotic fiber as a rather unspecific group of fermentable dietary fiber reduced CRP in 29 pooled RCTs. Combined pre- and probiotics (synbiotics) did not affect CRP but had a significant impact on TNF-alpha in 26 pooled trials [205]. Other meta-analyses on the impact of probiotics and synbiotics, i.e., specific bacteria alone or combined with fiber (usually inulin), did not point out an add-on effect of fiber [206,207].

### 5.3. Inulin

On the other hand, for inulin itself, anti-inflammatory properties are described in a recent meta-analysis. Supplementation decreases levels of CRP when pooling the eligible studies [208]. In T2DM patients, inulin also reduced IL-4, IL-12, and IFN-gamma after two months of supplementation [209]. In obese subjects, levels of calprotectin, an organ-specific marker of gut damage, were reduced by a combined 3-month treatment of inulin and hypocaloric diet (when compared to low-inulin hypocaloric diet), accommodated by changes in the gut microbiome and bacterial metabolites [210]. Several studies indicate that fermentation to short-chain fatty acids (acetate, butyrate, propionate) is somehow crucial for a part of the effect on inflammation markers [211,212]. In other studies on inulin, no anti-inflammatory effect was seen [213]. In patients with NASH, supplementation with a guar-inulin-symbiotic did not induce a differential effect on uric acid or ferritin levels, the only assessed inflammatory markers. However, despite randomization, the cohort distribution showed some lack of comparability between the treatment and control groups [214]. In summary, inulin seems to provide the same benefit as probiotics by using different points of action within the same mechanistic pathway.

### 5.4. Resistant Starch

Resistant starch type 2 (RS2), another type of soluble fiber, has been investigated in a series of RCTs. A meta-analysis pooling eight studies did not find a significant impact of RS2 on CRP, TNF-alpha, or IL-6 [215]. Two other SRMAs, pooling 13 or 16 studies, reported a benefit on TNF-alpha and IL-6, but not CRP [216,217]. A third SRMA detected a significant reduction in TNF-alpha, but neither IL-6 nor CRP [218]. In patients with end-stage renal disease, RS2 seems to lower IL-6, but not hsCRP, in five pooled RCTs [219]. A clinical effect of RS in patients with IBD is presumed, but study heterogeneity does not allow a strong support of supplementation [220].

### 5.5. Other Types of Soluble Fiber

Other types of soluble fermentable fiber are only sparsely investigated with respect to inflammation. Psyllium, administered over 7 weeks, did reduce IL-6 levels in overweight-to-obese adolescents, paralleling its effect on LDL cholesterol [221]. In another study on psyllium, supplementation of 7 and 14 g per day over 3 months improved fibrinogen levels but not CRP, IL-6, or leukocyte count [222]. In a later study, a naturally high-fiber DASH diet compared to a psyllium-supplemented diet induced a similar decrease in CRP levels, especially in normal-weight persons [223]. A total of 1,5 g of beta-glucans increased IL-10 levels after 4 weeks [224]. On the other hand, 6 g of oat beta-glucans over 6 weeks [225], 15 g of pectin over 3 weeks [226], and 10 g of guar gum over 6 weeks did not affect CRP levels despite their effects on LDL [227].

The overview, given in this chapter, suggests that most types of fiber do provide anti-inflammatory benefits. However, one needs to consider the enormous heterogeneity in study designs, involving intervention duration and dosage or the selected cohorts with their variable susceptibility to improvements or deterioration. Final conclusions on any type of fiber should not be drawn. In particular, this review section does not propagate fiber supplementation or fortification.

## 6. Putative Anti-Inflammatory Mechanisms of Dietary Fiber

**Figure 2:**
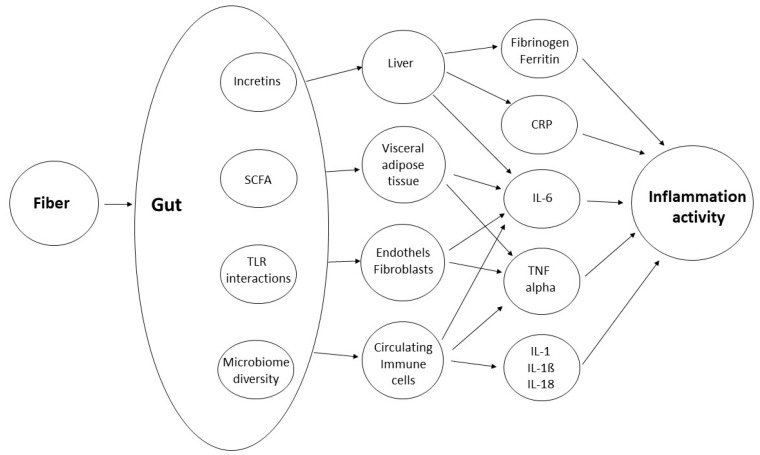
Fiber, gut and inflammatory mediators.

In order to extrapolate the magnitude of the anti-inflammatory effects of dietary fiber, one must understand the mechanisms behind that very action. Such effects could be related to nutrients besides fiber, which are consumed concomitantly (see above), but also to fiber itself. A graphical overview of the range of potential mechanisms is shown in Figure 2.

The inflammatory state in Metabolic Syndrome is mainly caused by visceral fat depots, which themselves trigger immune cell activity and the production of pro-inflammatory cytokines. To a smaller relative extent, subcutaneous fat mass contributes to systemic inflammation. Ergo, the most plausible mechanism of any anti-inflammatory treatment would be to reduce (visceral) fat mass in the process of weight loss [228]. However, there is inconsistent evidence that dietary fiber in general or specific types of fiber in particular promote a clinically relevant reduction in body fat [229]. In meta-analyses, high-fiber diets only support a very moderate weight loss. This is shown for whole grain, total fiber, and viscous soluble fiber, all providing a potential of roughly 0.5 kg fiber-driven weight reduction (irrespective of concomitant hypocaloric dietary regimens) [230,231], while more widely defined low-GI diets do not even lead to significant effects [21]. For polyglycoplex, glucomannan, resistant starch, and guar gum, there is also no consistent evidence for an impact on body weight regulation [232,233,234,235].

For certain types of fiber, direct effects on the immune system have been described. In various murine model experiments, pectin, fructanes, guar gum, and resistant starch—all being soluble and fermentable—elicit interactions with toll-like receptors, which lead to an anti-inflammatory response [236,237,238,239,240,241,242,243].

Other plausible means of action, by which dietary fiber reduces inflammatory signals, might be short-chain fatty acids, which are produced by bacterial fermentation in the gut. Clearly, this does not apply to non- or low-fermentable (typically cereal-type) fiber. SCFAs—this is acetate, propionate, and butyrate—are considered beneficial compounds both in the gut and after absorption. They reduce insulin resistance independent of body weight [146,147,244], feed certain strains of intestinal microbiota, and may stimulate the release of gastrointestinal hormones such as GLP-1 and PYY [147,245,246]. SCFAs also stimulate the G-protein-coupled receptors (GPR) GPR41 and GPR 43, which are located on mononuclear cells, but also adipose tissue. In rodent models, this signaling has been shown to modulate inflammatory state towards immunity and inflammatory homeostasis [247,248,249], but also to beneficially affect adipose tissue and blood vessels [250,251,252,253,254]. Microbiotic metabolism also leads to the production of other lipid compounds such as the immunomodulatory rumenic acid, as was shown for an inulin treatment in obese patients [210].

The impact of fiber on the gut microbiome extends way beyond the mere production of SCFAs. In patients with T2DM (but surely in other persons as well), treatment with soluble dietary fiber changed gut microbiome composition, in particular by promoting bifidobacteria. This is accommodated by lower exposition with lipopolysaccharides, which stimulate the host’s immune system [229,255]. Expansion of bifidobacteria is also connected to reduced levels of calprotectin, a gut-specific marker of inflammation [210].

Also, the gut microbiome interacts with tryptophane metabolites and bile acids, both of which co-regulate the intestinal immune system, the gut barrier, and post-absorptive liver function [256,257,258]. Bile acids are modulated by various types of fiber and elicit local effects on gut integrity and systemic effects on inflammation and metabolism, which are induced via the farnesoid X receptor (FXR) and the Takeda G-protein-coupled receptor (TGR) [259,260]. They are also involved in moderate insulin resistance following a high-protein diet, which can be counteracted with insoluble dietary fiber [261]. Induction of an increase in bile acids by insoluble fiber is apparently independent of fermentation, as it occurs with fermentable pea fiber, but also poorly fermentable cereal fiber [261,262]. Bile acids are a potential link between fiber intake, changes in gut microbial diversity, and effects on hepatic integrity (e.g., in the context of NAFLD) [263].

The effects of poorly fermentable cellulose can be explained in the context of certain rare bacterial genera that may actually cleave cellulose. The presence of cellulose allows microbial diversification that promotes an anti-inflammatory balance [264]. Augmentation of bacterial genera, which are capable of extensive polysaccharide degradation, has also been associated with a high intake of fruit fiber, in particular pectin [265]. In humans, a cellulose-hemicellulose supplement increased the fecal excretion of branched-chain amino acids (BCAAs), mirrored by elevated levels of fecal isovalerian acid but independent of fiber fermentation and changes in microbiota [200,266,267]. BCAAs promote adipose tissue activity, NAFLD, and insulin resistance via mTOR-S6K (mammalian target of rapamycin—S6 kinase) [268,269], and conversely, insulin resistance increases circulating BCAA levels by reduced amino acid metabolization [270,271].

Another potential mechanism of fiber action could be mediated by the glucose-dependent insulinotropic hormone (GIP). GIP primarily stimulates alpha- and beta-cells during glycemic excursions, leading to the secretion of glucagon and (thereby) insulin [272,273,274,275]. In periods with continuously elevated energy intake excess GIP secretion may lead to inflammatory processes, ectopic lipid storage (liver fat, visceral fat), and insulin resistance [276,277,278,279]. In rodents, inhibiting of GIP signaling prevents obesity and NAFLD [277,278,280,281,282]. There is also evidence for a pro-inflammatory effect in the hypothalamus [283]. GIP has been found to be acutely suppressed by rye and wheat whole grain [284,285,286,287,288,289,290], resistant starch [291,292,293,294,295], resistant dextrin [296], and guar gum [297]. Given the broad spectrum of effective interventions, including those based on poorly fermentable fiber, SCFAs do not seem to be the pivotal element in GIP suppression. Effects of fiber-rich diets on GIP secretion were also seen in animal models, where the general mechanistic connection between GIP and NAFLD could be confirmed. Consistently, resistant starch, cereal fiber, and various types of soluble fiber ameliorated glucose excursions and hormonal response to nutrient intake in rats [298,299,300,301] and pigs [302,303,304].

Understanding inflammation mechanisms and targeting them with specific treatments brings up a typical limitation of clinical practice—individual response. This may apply to complex diets, but also to well-defined specific food products or their active components. Even when controlling for circadian timing, healthy persons show an individually distinct glycometabolic response pattern to a variety of foods. This variability could be explained by differences in gut microbiome composition [305], while genetic variations, circadian rhythms, and concomitant behavioral aspects contribute to a highly diverse range of hormonal, metabolic, and inflammation-related responses [306,307,308].

## 7. Comprehension and Outlook

Many publications have assessed the effects of various types of fiber and fiber-rich food on the inflammatory system in humans, but studying heterogeneity does not allow consistent conclusions to be drawn. Fiber-rich foods reduced inflammation markers in almost all trials. Fruits and vegetables often seem to be less effective, maybe because of a concomitant sugar load. High-fiber diets may also just appear to be beneficial if additional components of the active comparator—such as PUFAs, vitamins, or phytate—are the actual players, or if prominent components of the control condition actually impair inflammation (iron, sugar, simple starch). Many studies examined the potential of synbiotic supplements, leaving the question of whether the isolated components would elicit a similar effect.

In well-controlled supplementation studies, some types of fiber, especially inulin and resistant starch, have been consistently shown to reduce inflammation. For highly fermentable fiber, the production of SCFAs seems to be a strongly contributing, but not a clearly mandatory component, in its mechanistic pathway. Insoluble fiber might elicit similar effects but is poorly investigated. It often resists fermentation, and different means of action are discussed.

Fiber-rich diets and—way less preferably—fiber supplementations may ameliorate the inflammatory axis of the Metabolic Syndrome supporting clinical improvement of chronic inflammatory disorders. However, the current state of the literature lacks sufficient evidence to address specific types of fiber, effective dosages, and indications. The current evidence encourages large-scale randomized trials for a variety of dietary fibers, targeting inflammatory outcomes in the context of metabolic disorders and beyond that. As a large body of existing evidence was generated by industry-funded projects, sufficient public funding is needed to reduce bias by potential conflicts of interest.

## Figures and Tables

**Table 1:** Chemicophysical diversity of fiber.

Type of Fiber	Water-Solubility	Fermentability	Viscosity	Monomers and Structure	Main Food Sources
Lignin	↓	↓	↓	Lignols, few hundred monomers	cereals and legumes, fruit stones
Cellulose	↓	↓	↓	β-(1-4)-linked glucose, unbranched, few thousand monomers	cereals and legumes
Cellodextrins	↑	↑	↓	β-(1-4)-linked glucose, unbranched, few hundred monomers	cereals and legumes
Chitin	↓	↓	↓	β-(1-4)-linked N-acetylglucosamine, unbranched	crustaceans, arthropods, mushrooms
Arabinoxylan	↓/↑	↓/↑	↓/↑	β-(1-4)-linked xylose, arabinose-branches, few hundred monomers	grains, psyllium
Arabinoxylan-oligosaccharides	↑	↓/↑	↑	β-(1-4)-linked xylose, arabinose-branches, few dozen monomers	grains, psyllium
Other pentose-Hemicelluloses	↓	↓	↓	β-(1-4)-linked pentoses, branched, few hundred monomers	oat, rye
Galactomannan	↑	↑	↑	β-(1-4)-linked mannose, galactose-branches, few hundred monomers	various gums (e.g., guar, cassia)
Other hexose-Hemicelluloses	↓	↓	↓	β-(1-4)-linked hexoses, branched, few hundred monomers	barley, wheat
Xyloglucan	↑	↓	↓	β-(1-4)-linked glucose, xylose-branches, few hundred monomers	fruits, vegetables
(mixed-linkage) ß-glucan	↑	↑	↑	β-(1-4)- β-(1-3)-linked glucose, few hundred monomers	barley, oat, rye
Resistant starch type 1	↓	↑	↓	α-(1-4)-linked glucose, α-(1-6)-linked branches, cellular matrix	unprocessed starchy vegetables
Resistant starch type 2	↓	↑	↓	α-(1-4)-linked glucose, α-(1-6)-linked branches, specific conformation	unripe fruits
Resistant starch type 3	↑	↑	↓	α-(1-4)-linked glucose, α-(1-6)-linked branches, retrograded	starchy, protein-containing foods
Resistant starch type 4	↑/↓	↑	↓	α-(1-4)-linked glucose, α-(1-6)-linked branches, chemically altered	synthetic alteration of starch
Resistant starch type 5	↑/↓	↑	↓	α-(1-4)-linked glucose, α-(1-6)-linked branches, in lipid complexes	processed starchy, fatty foods
Fructan (e.g., Inulin)	↑	↑↑	↑/↓	β-(2-1)- and/or β-(2-6)-linked fructose, unbranched, dozens of monomers	tubers and roots
Raffinoses	↑	↑	↑	(1-6)-linked oligosaccharides of galactose, fructose, glucose, unbranched	legumes, vegetables, grains
Pectin	↑	↑↑	↑	α-(1–4)-linked galacturonate, variable substitutes and branches	fruits and vegetables
Alginate	↑	↑	↑	(1-4)-linked mannuronate and guluronate, unbranched	brown seaweeds
Agar	↑	↑	↑	α-(1-3)/β-(1-4)-linked galactose and 3,6-anhydro-galactose, side-groups	red (and other) algae
Carrageenan	↑	↑	↑	sulfated (anhydro-)galactose in various linkage patterns, unbranched	red algae, food additive
Guar gum	↑	↑↑	↑	β-(1-4)-linked mannose with α-(1-6)-galactose side chains	guar, food additive
Xanthan	↑	↑	↑	β-(1-4)-linked glucose; glucose-mannose-glucuronate-branched	synthesized food additive
Polydextrose	↑	↑	↑	Glucose in variable α- and β-linkage; added by sorbitol and citric acid	synthesized food additive

Legend: ↑↑ = very high; ↑ = high; ↑/↓ = variable; ↓ = low.

## Data Availability

No new data were created or analyzed in this study.

## Abbreviations

CAM	cell-adhesion molecule
CRP	C-reactive protein
CVD	cardiovascular disease
GI	glycemic index
GIP	glucose-dependent insulinotropic peptide
GL	glycemic load
ICAM	intercellular cell adhesion molecule
IFG	impaired fasting glucose
IGT	impaired glucose tolerance
IL-1	interleukin 1
IL-1ß	interleukin 1 beta
IL-2	interleukin 2
IL-6	interleukin 6
IL-8	interleukin 8
IL-10	interleukin 10
MCP	monocyte chemoattractive protein
NAFLD	non-alcoholic fatty liver disease
RCT	randomized-controlled trial
RS	resistant starch
T2DM	type 2 diabetes mellitus
TNF-alpha	tumor-necrosis factor alpha
VCAM	vascular cell adhesion molecule
